# Successful identification of *Granulicatella adiacens* in postoperative acute infectious endophthalmitis using a bacterial 16S ribosomal RNA gene-sequencing platform with MinION™: A case report

**DOI:** 10.1016/j.ajoc.2022.101524

**Published:** 2022-04-12

**Authors:** Masato Ishino, Masatoshi Omi, Kaoru Araki-Sasaki, Shimpei Oba, Haruhiko Yamada, Yoshiyuki Matsuo, Kiichi Hirota, Kanji Takahashi

**Affiliations:** a Department of Ophthalmology, Kansai Medical University, Japan; b Department of Human Stress Response Science, Institute of Biomedical Science, Kansai Medical University, Japan

**Keywords:** Postoperative endophthalmitis, *Granulicatella adiacens*, Nutritionally variant *streptococci* (NVS), Nanopore sequencer, 16S rRNA gene analysis

## Abstract

**Purpose:**

To evaluate the efficacy of identifying the bacteria by aqueous sampling and vitreous sampling in postoperative infectious endophthalmitis using 16S ribosomal RNA (rRNA) gene analysis with a nanopore sequencer (MinION™).

**Observation:**

A 55-year-old woman who underwent cataract surgery at an ophthalmology clinic 18 days ago was referred to our hospital for suspected endophthalmitis. She had light perception visual acuity in her right eye; however, the eye was severely inflamed, with a hypopyon and a fibrinous membrane in the anterior chamber. The fundus was not visible because of vitreous opacity on a B-scan image. Based on the diagnosis of postoperative acute infectious endophthalmitis, we performed a vitrectomy, intraocular lens extraction, and silicone oil tamponade. On postoperative day 14, the inflammation resolved. An aqueous sample was collected before surgical treatment, and the vitreous sample was collected during the operation. Both samples underwent 16S rRNA gene analysis with a nanopore sequencer MinION™ to identify the causative organism.

**Conclusions and Importance:**

In the aqueous humor, *Granulicatella adiacens* and *Cutibacterium acnes* were identified before the operation, while only *Granulicatella adiacens* was detected in the vitreous sample after the operation. Although the aqueous humor sample might contain commensal bacteria, it could provide a predictable result before the operation. It can also provide a substitute for a vitreous sample to allow earlier identification of the causative organism.

## 1 Introduction

In the treatment of endophthalmitis, it is essential to differentiate whether the intraocular inflammation is infectious or non-infectious. In the case of infection, rapid identification of the causative organism is very important for further treatment. Usually, vitreous samples are collected at the time of vitrectomy, but aqueous humor can be collected before surgery in an outpatient setting, for earlier testing. We established a platform for the rapid identification of the causative organism using 16S rRNA gene analysis with the nanopore sequencer, MinION™ ^1^. Nanopore sequencing enables direct, real-time analysis of long DNA fragments through monitoring changes in the electrical current when nucleic acids pass through a nanopore. With this technology, there is no theoretical read length limit, and nanopore sequencing generates long reads that cover the entire region of the bacterial 16S rRNA gene. Full-length 16S rRNA gene sequencing provides high-resolution taxonomic analysis, which allows reliable and accurate representation of bacterial communities.

We report a 16S rRNA gene analysis with MinION™ using aqueous humor that successfully identified the causative organism within 4 h. Aqueous humor sampling with MinION™ can be used as a substitute for vitreous sampling.

## 2 Case report

A 55-year-old healthy woman underwent cataract surgery with intraocular lens implantation in her right eye at an ophthalmic clinic 18 days previously. She was referred to our hospital because of suspected endophthalmitis. Her right visual acuity was light perception, and her left visual acuity was 0.04 (1.2 x S-3.75D = C-0.75D Ax 160°). Intraocular pressure was 20 mmHg in both eyes. In the right eye, there was severe conjunctival and ciliary injection, severe hypopyon with fibrin in the anterior chamber, and a white mass in the lenticular capsule (Fig. 1A). The fundus of the right eye was not visible due to corneal edema or a hazy vitreous. B-scan ultrasonography showed dense vitreous opacity (Fig. 1B). Based on the suspicion of postoperative acute infectious endophthalmitis, the anterior chamber fluid was collected after disinfection with iodine. In addition, we performed a vitrectomy and intraocular lens extraction. During the operation, retinal hemorrhages, massive fibrin membranes, and occluded retinal veins were observed (Fig. 1C). A vitreous sample was collected during surgery. The vitreous cavity was irrigated with vancomycin (10 mg/mL) and ceftazidime (20 mg/mL) and at the end of the surgery and filled with silicone oil.

**Fig. 1A:**
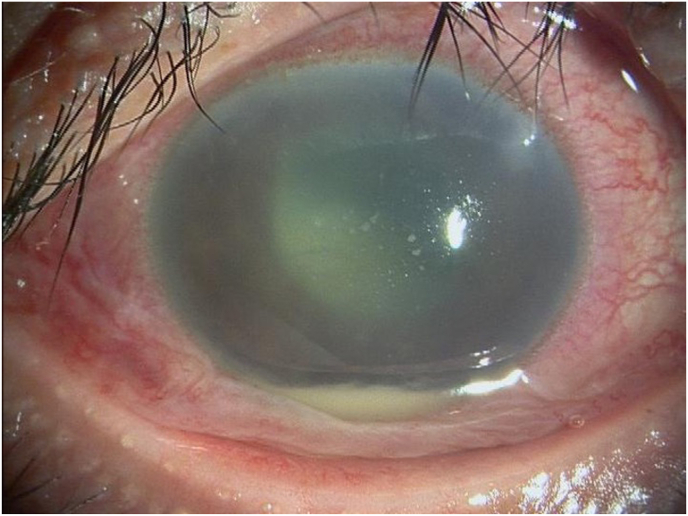
Photograph of the slit-lamp examination of the right eye during the first visit. Severe conjunctival and ciliary injection, stromal edema of the cornea, and white keratic precipitates with hypopyon were observed.

**Fig. 1B:**
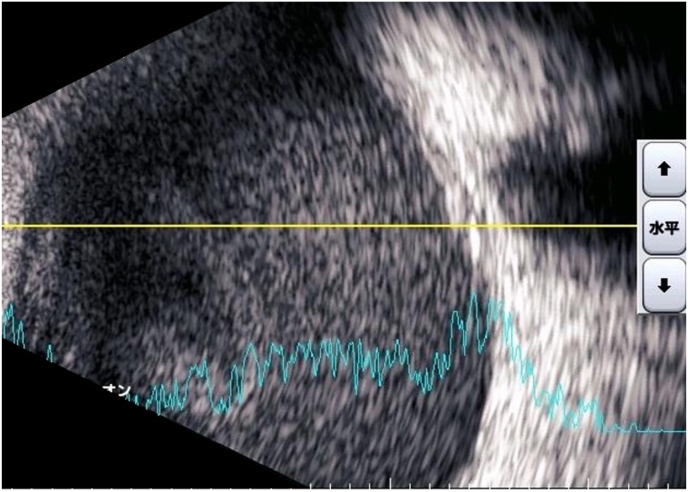
Ultrasound B-scan tomography of the right eye showed a reflection of dense vitreous opacity.

**Fig. 1C:**
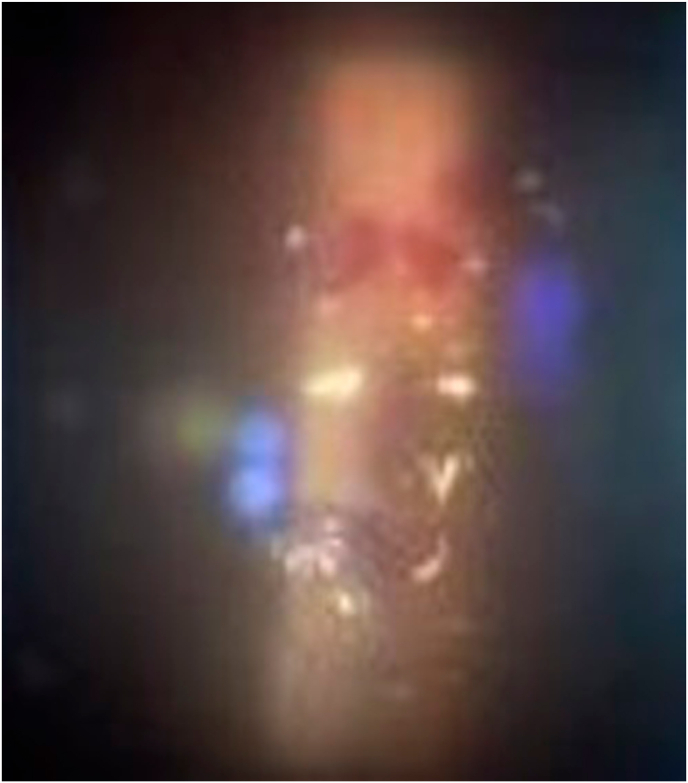
Retinal hemorrhages and occluded veins were identified intraoperatively.

We administered levofloxacin 1.5%, cefmenoxime 0.5%, and vancomycin eye drops to her right eye eight times per day, betamethasone 0.1% eye drops were applied to her right eye four times per day, and imipenem 1.0 g was administered intravenously twice per day, as an empiric therapy. On postoperative day 14, the injection was allowed to settle, and the fundus became visible. Approximately four months after the operation, the silicone oil was removed, and an intraocular lens insertion was performed. Although her corrected visual acuity in the right eye was 0.07 x S +5.00D = C −1.50D Ax 130°), the inflammation had completely resolved.

Vitreous and aqueous samples collected before and during vitrectomy were used for conventional culture with blood agar plates and 16S rRNA gene analysis. The extracted intraocular fibrous membrane was used for histological examination with hematoxylin and eosin (HE) staining and Gram staining.

No organisms grew on the blood agar plate from either the aqueous humor or the vitreous sample. HE staining of the fibrous membrane revealed numerous neutrophils (Fig. 2A), and Gram staining revealed some gram-positive cocci-like organisms. However, the microorganism identification was difficult (Fig. 2B), because the size and shape of the organism-like materials were heterogeneous. We performed 16S rRNA gene amplicon sequencing using the MinION™. The basic gene analysis protocol has been described previously,^1^ and a detailed protocol is also available at protocols.io (https://dx.doi.org/10.17504/protocols.io.bwr5pd86). *G. adiacens* and *Cutibacterium acnes* were detected in the aqueous humor by gene analysis, while only *G. adiacens* was identified in the vitreous sample (Fig. 3).

**Fig. 2A:**
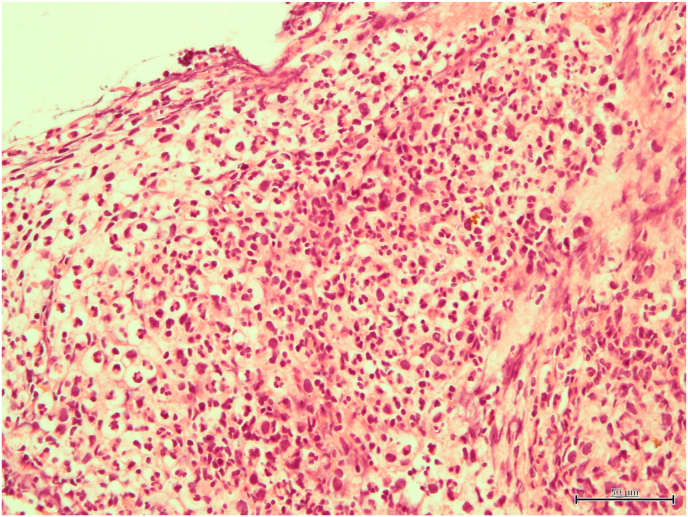
Numerous neutrophils were observed with HE staining of the fibrinous membrane obtained during surgery.HE, hematoxylin and eosin.

**Fig. 2B:**
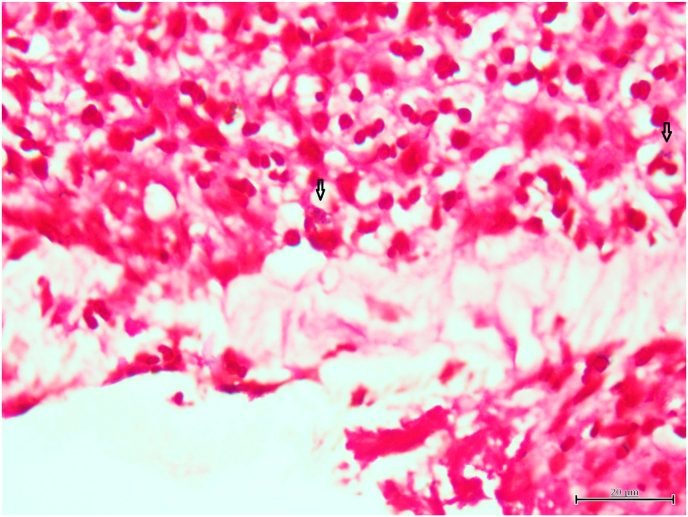
Gram staining of fibrinous tissue showed gram-positive cocci-like organisms phagocytosed by neutrophils.

**Fig. 3:**
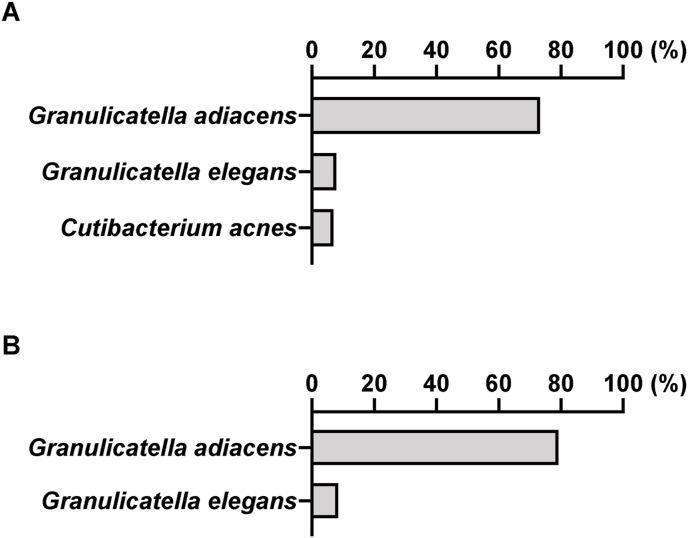
MinION™ sequencing of 16S ribosomal RNA (rRNA) gene amplicons identified *Granulicatella adiacens* in aqueous (A) and vitreous (B) samples. Results of MinION™ sequencing along with data analysis. Low-abundance taxa with less than 5% of classified reads were discarded from the analysis. *(A) Granulicatella adiacens* accounted for 88% and *Cutibacterium* acnes accounted for 6% of the identified bacterial species in the aqueous humor samples. *(B) Granulicatella adiacens* accounted for 95% of the identified bacterial species in the vitreous samples.

## 3 Discussion

In this report, we have shown that aqueous humor sampling can allow the rapid identification of the causative organism using 16S rRNA gene analysis with a nanopore sequencer. In the treatment of endophthalmitis, the rapid identification of the causative organism determines prognosis.

MinION™ offers real-time analysis of sequencing data, which enables the identification of pathogenic bacteria within a total turnaround time of 4 h. With this platform, it is meaningful to obtain aqueous humor before or at the beginning of the operation, because the identification of organisms can be obtained during or immediately following the surgery. The sequencing results allowed us to start an appropriate therapy soon after the surgery.

Recently, Jun et al.^2^ reported the microbial diagnosis of endophthalmitis using this device and verified an aqueous humor-based molecular diagnosis, also using this device, as a rapid, sensitive, and less invasive method for identifying causative pathogens. Our method differs in the primers that were used to identify bacteria and the kit used for library preparation. The selected kit may cause PCR product fragmentation during adapter addition, and our kit does not address this problem. Otherwise, our basic method was the same as that of Jun et al.^2^ The investigators emphasized that further case reports are needed to evaluate the clinical efficacy of this procedure, and the findings of our case report also warrant further investigation.

*Granulicatella* species have been reported to be associated with keratitis, conjunctivitis, orbital abscess, dacryocystitis, endophthalmitis, and ophthalmitis after intravitreal injection of anti-vascular endothelial growth factor agents.^3^, ^4^, ^5^, ^6^, ^7^, ^8^, ^9^, ^10^, ^11^, ^12^, ^13^, ^14^ The reports described difficulties in the detection of NVS using blood agar cultures. For example, Namdari et al.^9^ reported that blood agar supplemented with pyridoxal hydrochloride is necessary to isolate the organism. Concerning these organisms,16S-rRNA gene analysis with conventional next-generation sequencing has great potential for their detection, although the full analysis can take a few days. Endophthalmitis with negative culture includes cases where antimicrobial agents have already been administered, cases where the pathogen is localized, as in delayed onset endophthalmitis, and cases with organisms that are difficult to culture because of their high nutrient requirements, such as NVS. *G. adiacens*, an NVS, is difficult to culture and is responsible for culture-negative endophthalmitis.^15^^,^^16^ Todokoro et al. reported two cases of postoperative endophthalmitis caused by *G. adiacens* using 16S rRNA gene analysis.^7^ Subsequently, Pilli et al. described the clinical characteristics of endophthalmitis caused by *G. adiacens* using the same method.^8^ However, both identified the causative organism after surgery from the vitreous samples.

MinION™ sequencing involves a PCR amplification process that can simultaneously amplify indigenous bacteria. In fact, the anterior aqueous humor sample contained *Cutibacterium acnes*, which is considered an indigenous bacterium; however, the ratio of these indigenous bacteria was lower than that of the causative bacterium, *G. adiacens*, which was easily distinguished.

The rapid detection of *G.adiacens* in the anterior chamber aqueous humor or vitreous fluid by MinION™ will be a significant advantage in treating endophthalmitis. We listed the following four issues. (1) Digital identification can eliminate the risk of misdiagnosis of non-infectious endophthalmitis. (2) Confirmation of bacterial infection enables appropriate use of antimicrobial agents, including selecting the kinds and duration of administration. (3) Treatment can be possible without concern for fungi. (4) The possibility of aseptic endophthalmitis caused by vancomycin treatment can be ruled out.In our case, empiric therapy of antimicrobial agents was adopted in accordance with our endophthalmitis treatment path. This rapid and accurate identification method for *G. adiacens* using a next-generation sequencer can help establishment of a new treatment path that avoids the unnecessary administration of vancomycin and imipenem.

## 4 Conclusion

We succeeded in identifying the causative bacteria of infectious endophthalmitis from DNA extracted from anterior aqueous humor and vitreous samples using MinION™, a sequencer based on the nanopore principle. Identification of bacteria from the anterior atrial fluid may be useful in formulating treatment strategies and provides a substitute for a vitreous sampling to enable earlier identification of the causative organism.

## Patients consent

Presentation of this study was approved by the Ethics Committee of Kansai Medical University and the procedures conformed to the tenets of the Declaration of Helsinki. The patients consent was obtained by opt-out.

## CRediT statements

**Masato Ishino:** Writing- Original draft preparation, **Kaoru Araki-Sasaki:** Conceptualization, Methodology, Data analysis and Writing- Original draft preparation, **Masatoshi Omi and Shimpei Oba**: Gene analysis, Data curation, **Haruhiko Yamada**: Operation and Sample collection, **Yoshiyuki Matsuo and Kiichi Hirota**: Methodology, Draft Editing, Investigation and Supervision, **Kanji Takahashi**: Supervision.

## Funding

No funding was received for this work.

## Intellectual property

We confirm that we have given due consideration to the protection of intellectual property associated with this work and that there are no impediments to publication, including the timing of publication, with respect to intellectual property. In so doing we confirm that we have followed the regulations of our institutions concerning intellectual property.

## Research ethics

We further confirm that any aspect of the work covered in this manuscript that has involved human patients has been conducted with the ethical approval of all relevant bodies and that such approvals are acknowledged within the manuscript.

IRB approval was obtained (required for studies and series of 3 or more cases)

Written consent to publish potentially identifying information, such as details or the case and photographs, was obtained from the patient(s) or their legal guardian(s).

## Authorship

All listed authors meet the ICMJE criteria.  We attest that all authors contributed significantly to the creation of this manuscript, each having fulfilled criteria as established by the ICMJE.

## Contact with the editorial office

This author submitted this manuscript using his/her account in EVISE.

We understand that this Corresponding Author is the sole contact for the Editorial process (including EVISE and direct communications with the office). He/she is responsible for communicating with the other authors about progress, submissions of revisions and final approval of proofs.

We confirm that the email address shown below is accessible by the Corresponding Author, is the address to which Corresponding Author's EVISE account is linked, and has been configured to accept email from the editorial office of American Journal of Ophthalmology Case Reports:

## Declaration of competing interest

No conflict of interest exists.
